# Differential expression of estrogen receptor subtypes and variants in ovarian cancer: effects on cell invasion, proliferation and prognosis

**DOI:** 10.1186/s12885-017-3601-1

**Published:** 2017-08-31

**Authors:** Karen K. L. Chan, Michelle K. Y. Siu, Yu-xin Jiang, Jing-jing Wang, Yan Wang, Thomas H. Y. Leung, Stephanie S. Liu, Annie N. Y. Cheung, Hextan Y. S. Ngan

**Affiliations:** 1 Department of Obstetrics and Gynaecology, The University of Hong Kong, Queen Mary Hospital, Hong Kong, HKSAR China; 2 Department of Pathology, The University of Hong Kong, Queen Mary Hospital, Hong Kong, HKSAR China; 3 Department of Pathology, The University of Hong Kong - Shenzhen Hospital, Shenzhen, China

**Keywords:** Estrogen receptors, Ovarian cancer, Prognostic marker, Cell invasion and proliferation

## Abstract

**Background:**

Due to the presence of both classical estrogen receptor (ERα) and another ER subtype (ERβ) in ovarian cancer, hormonal treatment is an attractive option. However, response to tamoxifen in ovarian cancer is modest. The presence of ERβ variants further complicated the issue. We have recently shown that specifically targeting ER subtypes using selective ER modulators showed opposing functions of ER subtypes on cell growth. In the present study, the clinical significance of ERα and ERβ variants (β1, β2 and β5) and the functional effects of ERβ2 and ERβ5 in ovarian cancer was investigated.

**Methods:**

ERα, ERβ1, ERβ2 and ERβ5 expression were evaluated by immunohistochemistry in 106 ovarian cancer tissues. The association between ERs expression and clinicopathological parameters or prognosis was analyzed. Ectopic expression of ERβ2 and ERβ5 followed by functional assays were performed in ovarian cancer cell lines in order to detect their effects on cell invasion and proliferation.

**Results:**

We found significantly higher nuclear (n)ERα and nERβ5 and lower cytoplasmic (c)ERα expression in advanced cancers. Significantly lower ERβ1 expression was also detected in high grade cancers. Significant loss of nERα and cERβ2 expression were observed in clear cell histological subtypes. Higher nERβ5 and lower cERβ5 expression were associated with serous/clear cell subtypes, poor disease-free and overall survival. Positive cERα and higher cERβ1 expression were significantly associated with better disease-free and overall survival. Furthermore, we found nERβ5 as an independent prognostic factor for overall survival. Functionally, overexpression of ERβ5 enhanced ovarian cancer cell migration, invasion and proliferation via FAK/c-Src activation whereas ERβ2 induced cell migration and invasion.

**Conclusions:**

Since tamoxifen binds to both ERα and ERβ1 which appear to bear opposing oncogenic roles, the histotypes-specific expression pattern of ERs indicates that personalized treatment for women based on ERs expression using selective estrogen receptor modulators may improve response rate. This study also suggests nERβ5 as a potential prognostic marker and therapeutic target in ovarian cancer.

**Electronic supplementary material:**

The online version of this article (10.1186/s12885-017-3601-1) contains supplementary material, which is available to authorized users.

## Background

Ovarian cancer contributes to high mortality among all gynecological malignancies [1]. Primary treatment mainly involves cytoreductive surgery and adjuvant chemotherapy. Recurrences are common, albeit most patients have initial response. Thus, the overall prognosis is poor [2]. Although second line chemotherapy has overall 20–30% response rates, there are significant side effects. Hormonal therapy has relatively few side effects, making it as an attractive treatment option. Ovarian cancer is considered as a hormone-responsive cancer with estrogen receptors (ERs) expressed in about 60–100% of ovarian cancers [3]. Tamoxifen is a well-known selective estrogen receptor modulator (SERM) treatment for breast cancer. However, it only has a modest response rate (10–15%) in ovarian cancer [4]. It is crucial to unravel the way to make hormonal therapy more effective in ovarian cancer.

Estrogen acts via ERs. Another ER subtype (ERβ), which was discovered in 1996, was genetically different from the classical ERα [5, 6]. They differ not only in their tissue distribution, but also their ligand binding specificity and affinity [7]. We and others have found ERα and ERβ expression in normal and cancerous ovarian tissues [8, 9], with reduced ERβ expression when tumor progresses [8, 9]. Our recent study using ovarian cancer cell lines treated with specific SERMs showed opposing functions of ER subtypes on cell growth, suggesting specifically targeting ER subtypes using SERMs may offer women a new option when ER subtypes expression is known [10].

Besides subtypes, the presence of ERβ variants (β1-β5) due to alternative splicing further complicate the biological significance of ERβ signaling [11]. ERβ1 is the only isoform capable of binding ligands [11]. So, ERβ agonists and antagonists only bind ERβ1. ERβ3 is testis-specific [12]. Although ERβ2 and ERβ5 cannot bind ligands, they can heterodimerize with ERβ1 and induce its transcriptional activity ligand-dependently [11]. Differential expressions of ERβ1, ERβ2 and ERβ5 were found in colorectal, breast, endometrial and prostate carcinomas [13–16]. In prostate cancer, high ERβ2 expression was associated with poor prognosis [17].

Other than the classical genomic pathway, cytoplasmic ERs are also known to exert effects through non-genomic signaling [18]. In lung cancer cells, ERβ was found to have mainly non-genomic actions where ERβ was found in cytoplasm and could not translocate to the nucleus [19]. Moreover, ERβ2 has been found to be a significant prognostic marker in breast cancer with distinct outcome by nuclear and cytoplasmic expression, suggesting the importance of its subcellular functions [14].

A number of previous studies investigated prognostic roles of ERs in ovarian cancer, but the findings were controversial [8, 20–22]. A recent study found ERα is independent prognostic markers for endometrioid ovarian cancers [23]. Moreover, knowledge of ERs in ovarian caner with different histological subtypes is limited [3]. To the best of our knowledge, the present study is the only work assessing the subcellular expression of ERα, ERβ1, ERβ2 and ERβ5 in a well-validated cohort of different histotypes of ovarian cancers with complete follow-up data, using specific well-validated antibodies. The effects and downstream signaling of ERβ2 and ERβ5 on ovarian cancer cell invasion and proliferation were further investigated.

## Methods

### Clinical samples

One hundred and-six paraffin-embedded tissue blocks of ovarian cancer were obtained from Department of Pathology, University of Hong Kong, Queen Mary Hospital. All patients underwent surgery with the age range between 32 to 78 years (mean 50.2 years) and the follow-up period range between five to 209 months (mean 62 months). Seventy-six patients also received platinum/paclitaxel chemotherapy. To confirm diagnosis, all samples were histologically reviewed.

### Cell lines and subcellular protein extraction

Immortalized ovarian epithelial cell lines (HOSE 6–3, HOSE 11–12 and HOSE 17–1) and ovarian cancer cell lines (SKOV-3, OVCAR-3, OVCA 420, OVCA 429, OVCA 433, ES2, TOV-21G and TOV112D) were cultured as previously described [24, 25]. SKOV-3, OVCAR-3, ES2, TOV-21G and TOV112D were purchased from American Type Culture Collection (ATCC; Manassas, VA). Others were given by Prof. S.W. Tsao (Department of Anatomy, University of Hong Kong). Nuclear and cytoplasmic extracts from SKOV-3 cells were isolated as previously described [24, 25].

### Plasmids, transfection of ERβ2 and ERβ5, treatment with FAK inhibitor

Full-length sequences of *ERβ2* and *ERβ5* were assembled from synthetic oligonucleotides by GeneArt Gene Syntheses and cloned into pcDNA3.1 V5-His A (Life technologies, Waltham, MA). The final constructs were verified by sequencing and transfected along with the control vector into ES-2, OVCA420 and TOV-21G cells using Lipofectamine 3000 (Life technologies) and then selected with G418 (800 μg/ml) (Life technologies) [24, 25]. For FAK inhibitor treatment, ERβ5 overexpressing cells were plated 24 h before treating with the FAK inhibitor 14 (5 μM; Santa Cruz, Santa Cruz, CA) or vehicle (water). After 24 h, cells were harvested for immunoblotting.

### Immunohistochemistry

Immunohistochemistry was done on formalin-fixed, paraffin-embedded sections using EnVision + Dual Link System (K4061; Dako, Carpinteria, CA) as previously described [24, 25]. Antigen retrieval was done by heating in a pressure cooker with 1 mM EDTA (pH 8.0) (for ERα, ERβ1 and ERβ2) or citrate buffer (pH 6.0) (for ERβ5). Antigen were detected with antibodies against ERα, ERβ1, ERβ2 and ERβ5 (Additional file 1: Table S1). All four antibodies have been used/validated for immunohistochemical staining in paraffin-embedded tissue sections [14, 22]. Both the intensity (0 = negative, 1 = faint, 2 = moderate, and 3 = strong) and percentage (0 = <5%), 1 = 5%–25%, 2 = 26%–50%, 3 = 51%–75% and 4 = >75%) of stained epithelial cells were assessed semiquantitatively as previously described [24, 25]. A composite “Histoscore” was determined by multiplying the staining intensity by the percentage of stained cells with 12 as the maximum score. The “histoscores” cut off at mean was used to define high and low expression levels of target genes.

### Immunoblotting

Protein lysate was subjected to SDS-PAGE, transferred to PVDF membrane, and probed with antibodies as listed in Additional file 1: Table S1 and appropriate secondary antibodies as previously described [10, 24, 25]. Imaging of the bands were detected with ECL Plus detection system.

### Wound healing assay

ES-2 cells were seeded in six-well plates for 24 h. A wound was made by a sterile pipette tip. Photographs were taken at time 0 and 7 h to observe the closure of the wound as previously described [24].

### In vitro migration and invasion assays

Cells (1.25 × 10^5^) were plated on the upper side of a Transwell chamber (Corning, Tewksbury, MA) coated with or without Matrigel and then migrated or invaded through the membrane as previously described [24, 25]. After 7 (ES-2), 16 h (TOV-21G) or 24 h (OVCA420), cells on the upper compartment were removed. Migrated or invaded cells on the lower compartment were fixed, stained, and counted. For FAK inhibitor treatment, cells plated on the upper compartment for 6 h were treated with FAK inhibitor 14 (5 μM) or vehicle [24, 25].

### Cell count method, XTT assay and focus formation assay

For cell count method, cells (3 × 10^4^) were cultured in growth medium in 12-well or 6-well plates or T150 culture flasks as previously described [24]. After 24 h, cells were treated with 5 μM FAK inhibitor 14 or vehicle. Luna™ automated cell counter (Logos Biosystems, Annandale, VA) was used to count cell number at days 1 (12-well culture plates), 4 (6-well culture plates), 8 and 11 (T150 culture flasks) for ES-2 and days 1, 5, 9 and 11 for OVCA 420. For XTT assay (Roche), cells (2000 cells/well) were cultured in 96-well plates. 50 μl/well XTT labeling mixture was added at day 5. After 4 h incubation at 37 °C, cell viability was evaluated by assessing the absorbance at 492 nm.For focus formation assay, cells (2500) were seeded in 6-well culture plates and maintained in growth medium with fresh medium changed every 3 days. At day 9, cells were stained with 1% crystal violet (Sigma-Alrich). Numbers of foci were counted.

### Statistical analysis

SPSS 20 for Windows was used (SPSS Inc., Chicago, IL). Data between two groups was compared using Mann-Whitney test. Data among multiple groups was compared using Kruskal-Wallis rank test.For survival analysis, Kaplan–Meier analysis and log-rank test were done. For multivariate survival analysis, Cox regression analysis was performed. For correlation analysis, Spearman’s rho test was used. *P* values < 0.05 were considered statistically significant.

## Results

### Distinct subcellular localization patterns of ERs in ovarian cancers

**Fig. 1:**
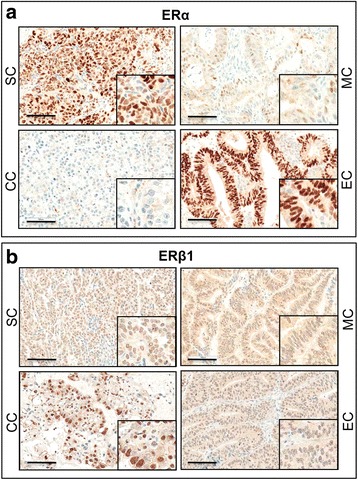
Immunohistochemical staining of ERα (**a**) and ERβ1 (**b**) in serous (SC), mucinous (MC), endometrial (EC) and clear cell (CC) carcinomas. Scale bar = 100 μm. *Insets* highlight regions with higher magnification

**Fig. 2:**
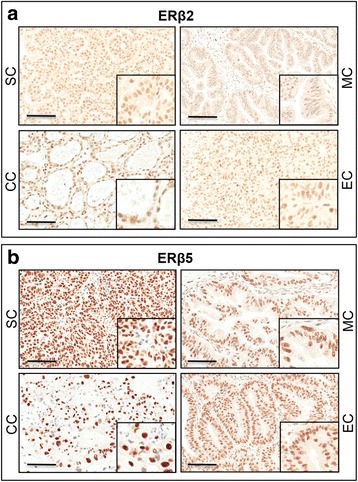
Immunohistochemical staining of ERβ2 (**a**) and ERβ5 (**b**) in serous (SC), mucinous (MC), endometrial (EC) and clear cell (CC) carcinomas. Scale bar = 100 μm. *Insets* highlight regions with higher magnification

By immunohistochemistry, we demonstrated distinct subcellular localization patterns of ERα, ERβ1, ERβ2 and ERβ5 in ovarian cancers (Figs. 1 and 2). Most of ERα (72%) was localized in the nucleus of ovarian cancers, but certain portion of ERα (16.7%) also resided in the cytoplasm (Fig. 1a). All ovarian cancers displayed all three ERβ variants in the nucleus, and 93, 96 and 68% of samples showing cytoplasmic ERβ1, ERβ2, and ERβ5 staining, respectively (Figs. 1b and 2). Moreover, nERβ1 (*P* = 0.041) immunoreactivities in metastatic foci was statistically lower than their corresponding primary carcinomas (Additional file 2: Figure S1).

### Correlation of ERs expression with clinicopathological parameters in ovarian cancer

To evaluate the clinicopathologic significance of ERs in ovarian cancer, “Histoscores” was analyzed with patients’ clinicopathologic parameters (Tables 1 and 2). Higher nERα (*P* = 0.012) and nERβ5 (*P* = 0.03) as well as lower cERα (*P* = 0.016) expressions were significantly associated with advanced stages (stages III-IV). Significantly lower cERβ1 (*P* = 0.034) expression was detected in stage IV carcinomas. Lower nERβ1 (*P* = 0.046) and cERβ1 (*P* = 0.046) expressions were significantly associated with poor histological differentiation (grade 3). nERα (*P* < 0.001) and cERβ2 (*P* = 0.001) expressions were significantly overexpressed in non-clear cell histological subtypes. Significantly higher nERβ1 (*P* = 0.003) and nERβ5 (*P* = 0.039) as well as lower cERβ5 (*P* = 0.013) expressions were found in serous/clear cell histological subtypes.

**Table 1:** Correlation of nucleus and cytoplasmic ERα and ERβ1 with clinicopathological parameters in ovarian cancer

Characteristics	Case (n)	Nucleus ERα			Cytoplasmic ERα			Nucleus ERβ1			Cytoplasmic ERβ1		
		Mean ± SD	*p*-value		Mean ± SD	*p*-value		Mean ± SD	*p*-value		Mean ± SD	*p*-value	
Stage (FIGO)													
Early (I-II)	49	3.71 ± 3.32			1.00 ± 1.75			5.60 ± 1.94			3.65 ± 1.67		
Late (III-IV)	37	5.54 ± 3.12	0.012 ^*^	↑	0.22 ± 0.92	0.016 ^*^	↓	6.35 ± 1.64	0.052^*^		2.84 ± 1.39	0.101^*^	
Histological grade													
Low (1–2)	56	4.11 ± 3.48			0.80 ± 1.62			6.25 ± 1.97			3.39 ± 1.67		
High (3)	33	4.85 ± 3.08	0.370^*^		0.36 ± 1.17	0.178^*^		5.42 ± 1.68	0.046 ^*^	↓	2.79 ± 1.43	0.046 ^*^	↓
Histology													
Serous	35	5.43 ± 3.15			0.34 ± 1.14			6.40 ± 1.94			2.91 ± 1.46		
Clear Cell	17	1.06 ± 2.02			0.71 ± 1.58			6.76 ± 1.64			3.06 ± 1.78		
Mucinous	9	2.22 ± 2.73			0.44 ± 1.33			5.56 ± 2.46			3.44 ± 2.40		
Endometrioid	29	5.90 ± 2.68	< 0.001 ^†^		1.10 ± 1.82	0.230^†^		5.20 ± 1.54	0.021 ^†^		3.40 ± 1.35	0.567^†^	
Serous/Clear Cell	52	4.00 ± 3.49			0.46 ± 1.29			6.52 ± 1.84			2.96 ± 1.56		
Mucinous/Endometrioid	38	5.05 ± 3.09	0.143^*^		0.95 ± 1.72	0.129^*^		5.28 ± 1.76	0.003 ^*^	↓	3.41 ± 1.62	0.157^*^	
Clear Cell	17	1.06 ± 2.02			0.71 ± 1.58			6.76 ± 1.64			3.06 ± 1.78		
Non-Clear Cell	73	5.23 ± 3.10	< 0.001 ^*^	↑	0.66 ± 1.49	0.905^*^		5.81 ± 1.92	0.053^*^		3.18 ± 1.56	0.593^*^	
Chemosensitivity^a^													
Sensitive	63	4.64 ± 3.12			0.76 ± 1.58			6.12 ± 1.93			3.14 ± 1.70		
Resistant	13	4.38 ± 3.97	0.885^*^		0.31 ± 1.11	0.325^*^		5.85 ± 1.46	0.609^*^		2.77 ± 0.73	0.409^*^	

Those with significant *P*-values are underlined. ↑Increase expression. ↓Decrease expression

^*^Mann–Whitney test; ^**†**^Kruskal–Wallis rank test

^a^Chemosensitive-patients remained disease free more than 6 months after completion of first-line chemotherapy

Intensity values are expressed as “Histoscores” as specified in Methods

**Table 2:** Correlation of nucleus and cytoplasmic ERβ2 and ERβ5 with clinicopathological parameters in ovarian cancer

Characteristics	Case (n)	Nucleus ERβ2		Cytoplasmic ERβ2			Nucleus ERβ5		Cytoplasmic ERβ5			
		Mean ± SD	*p*-value	Mean ± SD	*p*-value		Mean ± SD	*p*-value		Mean ± SD	*p*-value	
Stage (FIGO)												
Early (I-II)	49	5.46 ± 1.50		3.90 ± 1.43			6.61 ± 1.10			1.86 ± 1.67		
Late (III-IV)	37	5.65 ± 1.60	0.936^*^	3.78 ± 1.40	0.701^*^		7.11 ± 1.22	0.030 ^*^	↑	1.46 ± 1.77	0.165^*^	
Histological grade												
Low (1–2)	56	5.49 ± 1.76		3.84 ± 1.58			6.88 ± 1.06			1.70 ± 1.72		
High (3)	33	5.64 ± 1.27	0.996^*^	3.88 ± 1.32	0.784^*^		6.82 ± 1.29	0.751^*^		1.67 ± 1.80	0.904^*^	
Histology												
Serous	35	5.80 ± 1.68		4.06 ± 1.24			6.89 ± 1.37			1.40 ± 1.77		
Clear Cell	17	5.71 ± 1.26		3.00 ± 2.00			7.29 ± 1.11			1.06 ± 1.44		
Mucinous	9	4.63 ± 2.26		3.25 ± 1.49			6.22 ± 0.67			1.78 ± 1.64		
Endometrioid	29	5.40 ± 1.35	0.608^†^	4.27 ± 1.14	0.005 ^†^		6.70 ± 0.92	0.079^†^		2.33 ± 1.73	0.071^†^	
Serous/Clear Cell	52	5.77 ± 1.54		3.71 ± 1.59			7.02 ± 1.29			1.29 ± 1.66		
Mucinous/Endometrioid	38	5.24 ± 1.58	0.237^*^	4.05 ± 1.27	0.246^*^		6.59 ± 0.88	0.039 ^*^	↓	2.21 ± 1.70	0.013 ^*^	↑
Clear Cell	17	5.71 ± 1.26		3.00 ± 2.00			7.29 ± 1.11			1.06 ± 1.44		
Non-Clear Cell	73	5.51 ± 1.64	0.580^*^	4.05 ± 1.25	0.001 ^*^	↑	6.73 ± 1.14	0.095^*^		1.82 ± 1.77	0.134^*^	
Chemosensitivity^a^												
Sensitive	63	5.56 ± 1.57		3.64 ± 1.36			6.84 ± 1.13			1.67 ± 1.72		
Resistant	13	5.92 ± 1.38	0.595^*^	4.46 ± 1.66	0.105^*^		6.92 ± 1.12	0.387^*^		1.69 ± 1.93	0.846^*^	

Those with significant *P*-values are underlined. ↑Increase expression. ↓Decrease expression

^*^Mann–Whitney test; ^**†**^Kruskal–Wallis rank test

^a^Chemosensitive-patients remained disease free more than 6 months after completion of first-line chemotherapy

Intensity values are expressed as “Histoscores” as specified in Methods

### Association between ERs expression and clinical outcome

**Fig. 3:**
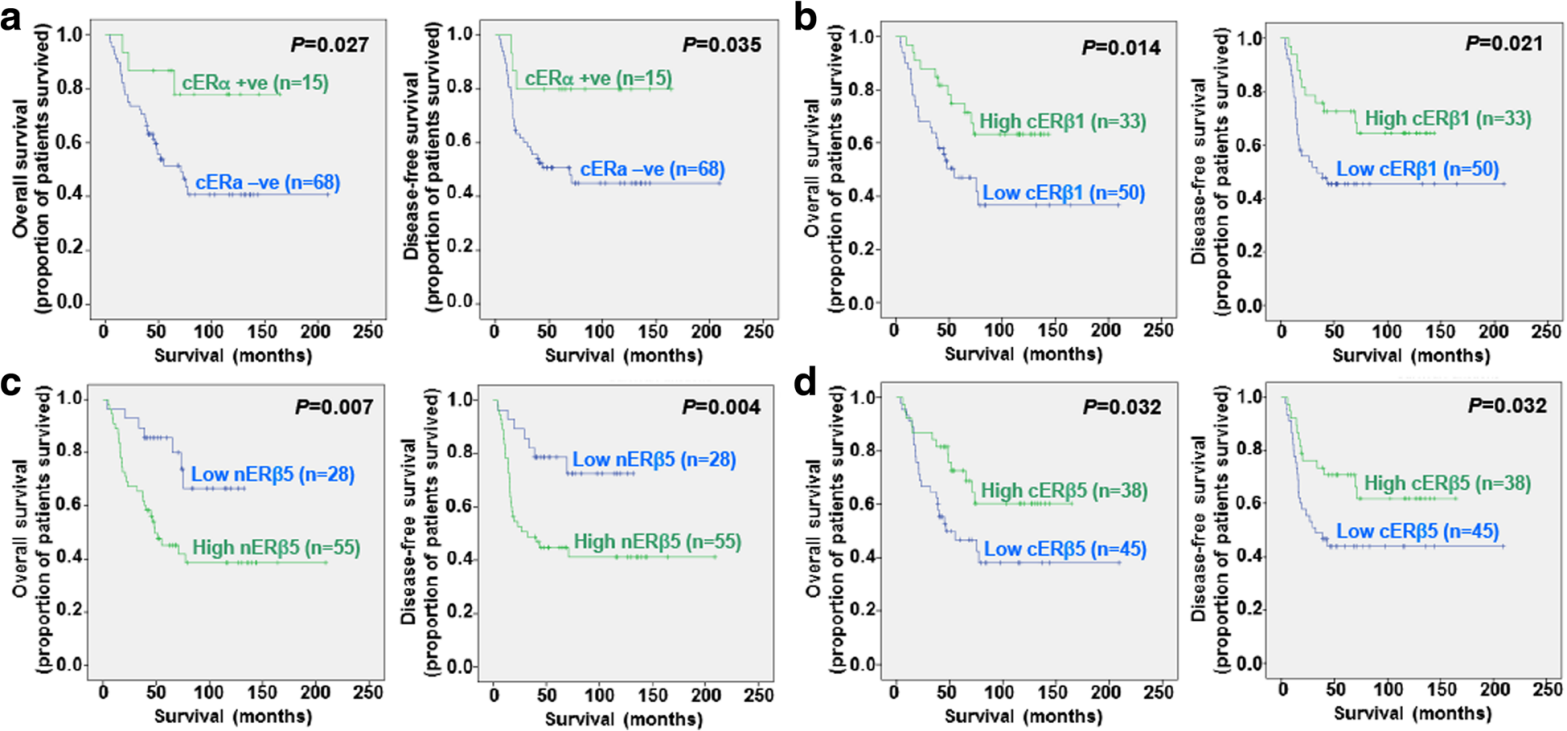
Kaplan-Meier overall (left panel) and disease-free (right panel) survival curves for ovarian cancer patients with positive (histoscores > 0) and negative cERα (**a**) expression, and high and low levels of cERβ1 (**b**), nERβ5 (**c**) and cERβ5 (**d**) (cut off at mean)

Univariate Kaplan-Meier-survival analysis demonstrated better overall and disease-free survival for cERα positive (*P* = 0.027 and *P* = 0.035; Fig. 3a) and high cERβ1 expression (*P* = 0.014 and *P* = 0.021; Fig. 3b) ovarian cancers. Interestingly, we found inverse relation between nuclear and cytoplasmic ERβ5 with survival. Significantly association was detected in high nERβ5 (*P* = 0.007 and *P* = 0.004; Fig. 3c) and low cERβ5 (both *P* = 0.032; Fig. 3d) expression with poor overall and disease-free survival. For overall survival, nERβ5, stage and chemosensitivity were significant predictors by multivariate analysis (all *P* < 0.05, Table 3).

**Table 3:** Cox regression analysis for factors affecting overall survival

Prognostic factor	Overall survival		
	*P*	Hazard Ratio	95% Confidence Interval
Nucleus ERβ5	0.024	3.297	1.169–9.303
Disease stage	0.008	3.831	1.411–10.402
Chemosensitivity	< 0.001	12.984	4.681–36.011

### ERs correlations

Spearman’s ρ test was performed to find correlations between ERs in ovarian cancers (Additional file 3: Table S2). nERβ1 correlated directly with cERβ1 (*P* = 0.003), nERβ5 (*P* = 0.038). cERβ1 correlated directly with cERβ2 (*P* = 0.008) and cERβ5 (*P* = 0.001).

### Differential expression of ERs in three normal HOSE and eight ovarian cancer cell lines and their subcellular expression in SKOV-3 cells

**Fig. 4:**
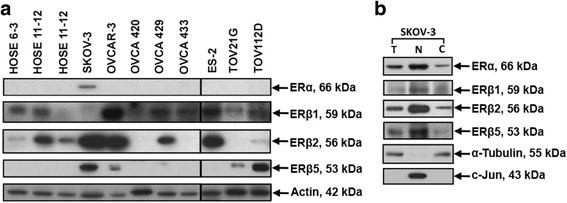
**a** ERα, ERβ1, ERβ2 and ERβ5 expression in immortalized human normal ovarian epithelial cell lines, HOSE 6–3, HOSE 11–12, HOSE 17–1 and ovarian cancer cell lines, SKOV-3, OVCAR-3, OVCA 420, OVCA 429 and OVCA 433, ES2, TOV-21G, TOV112D as determined by immunoblotting. Protein samples were resolved in two gels at the same time. **b** ERα, ERβ1, ERβ2 and ERβ5 in subcellular protein fractions of SKOV-3 (T: total cell lysate, N: nuclear fraction, C: cytoplasmic fraction)

By immunoblotting, ERα was found in SKOV-3, but not in HOSE cell lines and other cancer cell lines (Fig. 4a). Similar expression of ERβ1 was detected in both normal and cancer cell lines. Higher ERβ2 expression was shown in SKOV-3, OVCAR-3, OVCA 429 and ES2 than HOSE 6–3. ERβ5 expression was demonstrated in SKOV-3, OVCAR-3, OVCA 429, TOV-21G and TOV112D, but not in HOSE cell lines. Western blot analysis revealed subcellular expression of ERα, ERβ1, ERβ2 and ERβ5 in nuclear and cytoplasmic fractions of SKOV-3 (Fig. 4b).

### Overexpression of ERβ5 increased ovarian cancer cell invasion and proliferation in association with induced FAK activation

**Fig. 5:**
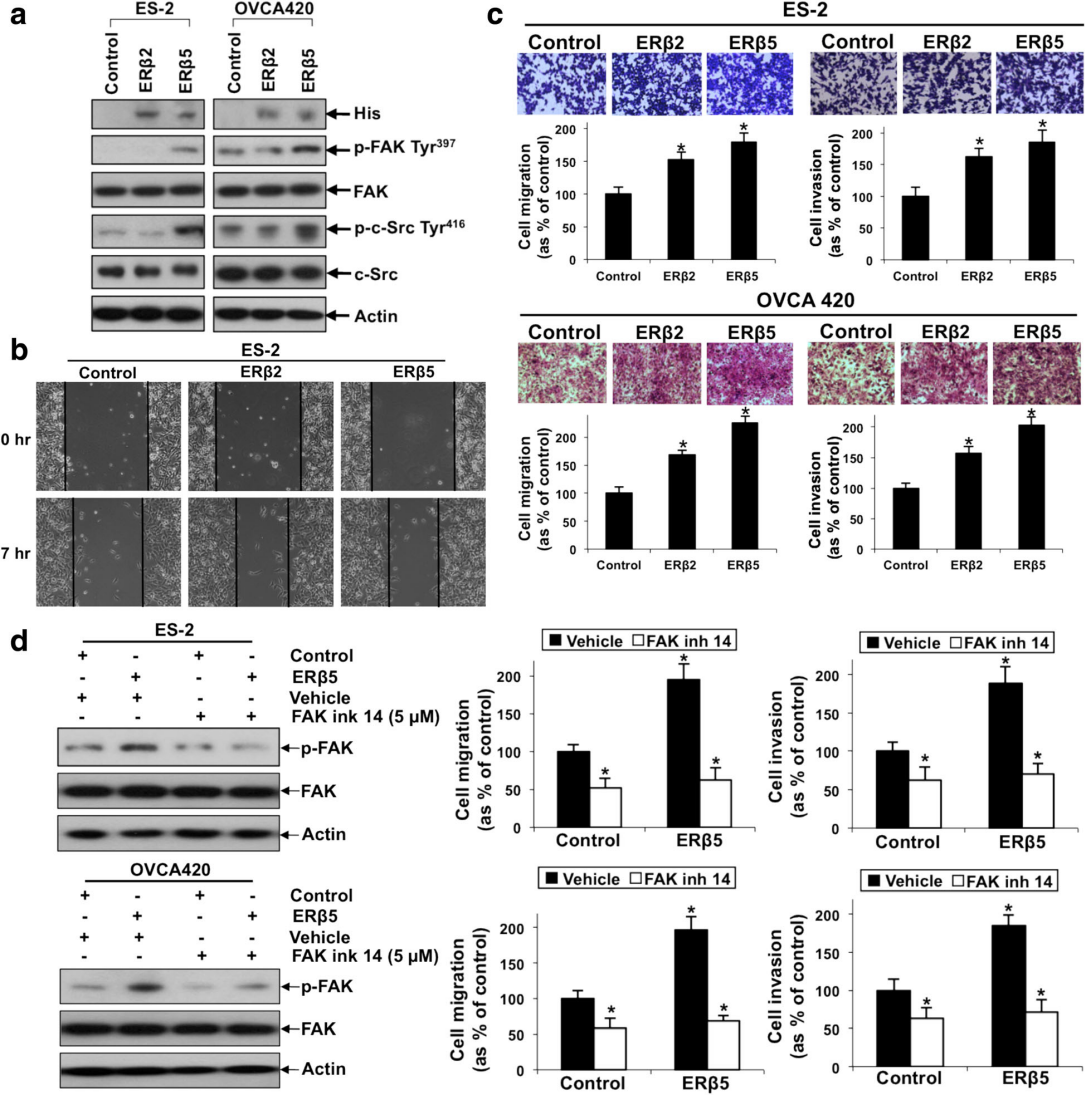
Overexpression of ERβ2 and ERβ5 increased ovarian cancer cell migration and invasion. ERβ5-mediated ovarian cancer cell migration and invasion involved FAK activation. **a** Immunoblot analyses of exogenous His-tagged ERβ2 and ERβ5, p-FAK Tyr^397^, FAK, p-c-Src Tyr^416^ and c-Src in ES-2 (left panel) and OVCA420 (right panel) cells stably transfected with His-tagged ERβ2, ERβ5 or control vector. **b** Wound healing assay and **c** in vitro migration and invasion assays in ES-2 and OVCA420 cells overexpressing ERβ2 and ERβ5. Upper panel: representative images of migrating or invading cells. Lower panel: Cell migration or invasion presented as percentage of control; *n* = 3; *, *p* < 0.05. **d** Left panel: immunoblot analysis on p-FAK Tyr^397^ and FAK in ES-2 and OVCA420 cells overexpressing ERβ5 in the presence or absence of FAK inh 14 or water (vehicle). Right panel: in vitro migration and invasion assays in ERβ5 overexpressing ES-2 and OVCA420 cells in the presence or absence of FAK inh 14 or water. Cell migration or invasion presented as percentage of control; *n* = 3; *, *p* < 0.05

Stable overexpression of ERβ2 and ERβ5 in ES-2 and OVCA420 was detected by immunoblotting using His-Tag antibody (Fig. 5a). To further verify the specificity of ERβ2 and ERβ5 antibodies, immunoblotting was performed on OVCA420 cells after stable overexpression of ERβ2 and ERβ5. By using anti-ERβ2 and anti-ERβ5 antibodies, increased expression of ERβ2 and ERβ5 was detected (Additional file 4: Figure S2). Then, we examined the roles of ERβ2 and ERβ5 on cell migration and invasion. ES-2 stably transfected with ERβ2 and ERβ5 displayed a faster migration rate when compared to control cells by a wound healing assay (Fig. 5b). Significantly increased migration and invasion (*P* < 0.05) in ERβ2 and ERβ5 overexpressing ES-2 and OVCA420 cells was demonstrated by Transwell migration and invasion assays (Fig. 5c). Significantly increased migration and invasion in ERβ2 overexpressing TOV-21G cells was also detected (*P* < 0.05) (Additional file 5: Figure S3).

**Fig. 6:**
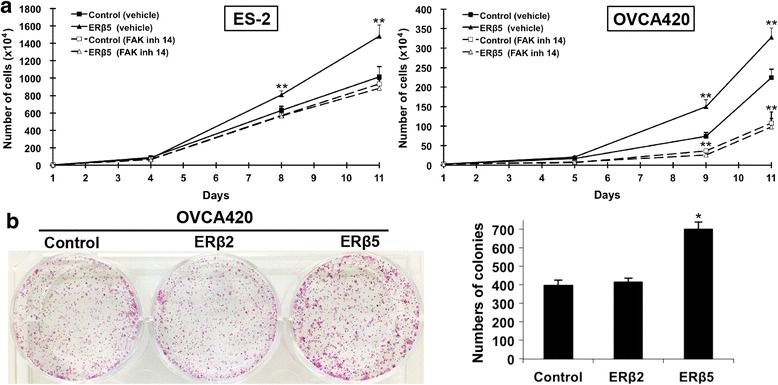
Overexpression of ERβ5 increased ovarian cancer cell proliferation and involved FAK activation. **a** Cell proliferation rate of ERβ5 overexpressing ES-2 (left panel) and OVCA420 (right panel) cells in the presence or absence of FAK inh 14 or water. **, *P* < 0.005. **b** A focus formation assay in OVCA420 overexpressing ERβ2 and ERβ5 presented as number of colonies formed. *n* = 3; *, *p* < 0.05

Next, we examined the possible downstream pathway of ERβ5. Focal adhesion kinase (FAK) and c-Src are key components of cell-matrix adhesion complexes, thus play important roles on cancer cell migration, invasion and metastasis [26]. We found ERβ5, but not ERβ2, induced FAK and c-Src activities as detected by phosphorylation on Tyr^397^ and Tyr^416^, respectively (Fig. 5a). To investigate the involvement of ERβ5-induced FAK activity on cell migration and invasion, overexpressing ERβ5 ES-2 and OVCA420 cells were treated with a FAK inhibitor (5 μM FAK inh 14). We found that FAK inh 14 could inhibit FAK activation (Fig. 5d) and abolish not only basal, but also ERβ5-induced cell migration and invasion (Fig. 5d). We next investigated the effects on cell proliferation. By cell count method, ERβ5 significantly induced cell proliferation in ES-2 and OVCA420 cells after 8 and 9 days respectively (Fig. 6a), albeit no significant increase of cell proliferation on day 5 (early time point) as assessed by XTT assay (Additional file 6: Figure S4). By focus formation assay, the number of colonies from OVCA420 cells overexpressing ERβ5, but not ERβ2, increased by about 75% (Fig. 6b). Besides metastasis, FAK also promotes cell proliferation [27]. Thus, we sought to examine if ERβ5-induced FAK activation could affect cell proliferation. Intriguingly, FAK inh 14 (Fig. 6a) blocked the ERβ5-mediated increase in ES-2 and OVCA420 cell proliferation (Fig. 6a). FAK inh 14 also inhibited OVCA420 basal cell proliferation (Fig. 6a and Additional file 6: Figure S4).

## Discussion

In the present study, we have shown ERα, ERβ1, ERβ2 and ERβ5 expression in nucleus and cytoplasm of ovarian cancer cells. ERs classically mediate their effects by genomic pathway [18]. Our recent study has documented decreased cell growth in ERα/ERβ1-expressing ovarian cancer cells, SKOV3 and OV2008, treated with MPP (ERα antagonist) and enhanced cell growth after treated with PPT (ERα agonist) [10]. An in vivo study also demonstrated that E2 significantly enhanced tumor size and promoted lymph node metastasis in ER^+^ ovarian tumors [28]. These findings together with our present data showing higher nERα expression in advanced stages of disease suggested an aggressive role of E2/nERα signaling in ovarian cancer. Cytoplasmic ERs are also known to exert effects through non-genomic signaling, which may involve cross-talk with other growth-factor receptors or cytoplasmic kinases [18]. Specific cytoplasmic ERα staining has been detected in breast cancer clinical samples using multiple validated antibodies, albeit the average incidence was only 1.5% [29]. This study has validated multiple antibodies including the one that bind to the “SP1” epitope [29]. The present study using an antibody that recognizes “SP1” epitope also detected both nuclear and cytoplasmic staining in ovarian cancer clinical samples. We found a significant correlation between positive cERα immunoreactivity and longer disease free and overall survival. Thus cERα could be a potential prognostic marker in ovarian cancer. A recent study showed that extranuclear ERα was involved in the regression of tamoxifen-resistant PKCα-overexpressing breast tumors [30]. It is possible that cERα plays anti-oncogenic roles in ovarian cancer which will be studied in near future.

This study revealed lower nERβ1 immunoreactivity in 16 metastatic foci than their paired primary cancers, suggesting that loss of nERβ1 may contribute to ovarian cancer metastasis. This was in agreement with previous findings where overexpression of ERβ1 was shown to repress in vitro cell migration and invasion in ovarian cancer cells [31, 32] as well as reduce tumor formation in sites of metastasis in vivo [33]. Besides cell migration, ectopic overexpression of ERβ1 also inhibited proliferation of ovarian cancer cells which was accompanied by induced p21, a cyclin-dependent kinase inhibitor, and reduced cyclin A2 mRNA expressions [31, 34]. Moreover, we recently reported that ovarian cancer cells treated with DPN (ERβ1 agonist) suppressed cell growth in vitro and in vivo and was accompanied by inhibition of phosphorylation of AKT, a non-genomic signaling pathway [10]. All these findings together with our present data showing lower immunoreactivity of cERβ1 in advanced carcinomas and poor histological differentiation as well as correlation with poorer survival further support that ERβ1 present in the cytoplasm functions as a tumor suppressor in ovarian cancers [20, 35].

We also showed significantly higher nERβ5 immunoreactivity in late stage disease and serous and clear cell histological subtypes. These findings suggest that nERβ5 affects the aggressiveness of the disease. Furthermore, a significant correlation between high nERβ5 immunoreactivity and poorer survival demonstrated nERβ5 as a potential prognostic marker in ovarian cancer. In contrast to nERβ5, we demonstrated cERβ5 as a favorable prognostic marker in ovarian cancer. We further found lower cERβ5 immunoreactivity in late stage disease. In non-small cell lung cancer, a study also documented cERβ5 to be negatively correlated with pathological stage and predicted long overall and disease-free survival [36]. Our data suggested that while nERβ5 may have an oncogenic role in ovarian cancer, cERβ5 may have anti-oncogenic role. Studies on the functional roles of ERβ2 and ERβ5 in cancers are limited. ERβ5 in breast cancer cells has been found to enhance apoptosis induced by chemotherapeutic agent through Bcl2L12 interaction [37]. In prostate cancer cells, ERβ5 increased cell migration and invasion [16]. A recent study has demonstrated antiapoptotic function of ERβ2 in advanced serious ovarian cancer [38]. In this study, we presented the first time the cell migration, invasion and proliferation enhancement roles of ERβ5 in ovarian cancer cells. FAK, a cytoplasmic protein tyrosine kinase, has been shown to be overexpressed and activated in numerous solid cancers and is linked to poor prognosis including in ovarian cancer [39]. In preclinical studies, FAK inhibitors inhibited tumor growth and metastasis. A safe and well-tolerated FAK inhibitor has also been reported in a clinical trial study [39]. Moreover, activated FAK can form complex and activate c-Src [39]. Our present study demonstrated that ERβ5-induced cell migration, invasion and proliferation may involve FAK/c-Src activation in ovarian cancer. nERβ5 may have an oncogenic role, wherease cERβ5 may have anti-oncogenic role in ovarian cancer, yet, we detected activation of cytoplasmic tyrosine kinases FAK/c-Src by ERβ5. It is possible that the activation of FAK/c-Src is an indirect activation via nERβ5 target genes, which will be studied in near future. Unlike ERβ5, ERβ2 was shown to affect ovarian cell migration and invasion, but not proliferation. It would be worthy to investigate the downstream target regulating ERβ2-induced ovarian cancer cell migration and invasion in future study.

Interestingly, the present study demonstrated differential ER subtypes and variants expression in different histological types of ovarian cancer. nERα was barely detectable in clear cell histological subtype. Such observation has been reported by others and loss of ERα in clear cell tumor was related to hypermethylation [40, 41]. We further detected significantly higher nERβ1 and nERβ5 as well as lower cERβ5 in serous/clear cell histological subtypes. Moreover, nERβ1 positively correlated with nERβ5 whereas cERβ1 positively correlated with cERβ5, suggesting ERβ1 and ERβ5 maybe tightly regulated. A recent Ovarian Tumor Tissue Analysis consortium study also revealed association between ERα expression and histotype-specific survival. ERα is an independent prognostic marker for endometrioid ovarian cancers [23].

## Conclusions

There are now increasing evidence to suggest that targeting individual ER subtypes by new SERMs with different ERα/ERβ1 binding affinities can maximize the hormonal response [3, 10, 42]. The differential ERα and ERβ1 expression in ovarian cancer and in different histological types as shown in the present study may help to explain the poor response rate of tamoxifen (10–15%) in ovarian cancer because tamoxifen binds to both ERα and ERβ1 and most clinical studies using tamoxifen therapy included patients with all histotypes [43, 44]. Moreover, our findings showed ERβ5 plays an important role in ovarian tumorigenesis by regulating cell migration, invasion and proliferation via FAK/c-Src activation. This study also suggests nERβ5 as a potential prognostic marker and therapeutic target in ovarian cancer.

## Additional files

Additional file 1: Table S1. Primary antibodies used for immunohistochemistry and immunoblotting. (DOC 43 kb)

Additional file 2: Figure S1. Box plot showing comparison of the median nERβ1 immunoreactivity score in primary carcinomas versus matched metastatic foci. (PDF 69 kb)

Additional file 3: Table S2. Correlation coefficients between ERs expression in ovarian cancer. (DOC 55 kb)

Additional file 4: Figure S2. Immunoblot analyses of ERβ2 and ERβ5 in OVCA420 cells stably transfected with His-tagged ERβ2, ERβ5 or control vector. (PDF 57 kb)

Additional file 5: Figure S3. Overexpression of ERβ2 increased ovarian cancer cell migration and invasion. (a) Immunoblot analyses of exogenous His-tagged ERβ2 in TOV-21G cells stably transfected with His-tagged ERβ2 or control vector. (b) In vitro migration and invasion assays in TOV-21G cells overexpressing ERβ2. Upper panel: representative images of migrating or invading cells. Lower panel: Cell migration or invasion presented as percentage of control; *n* = 3; *, *p* < 0.05. (PDF 223 kb)

Additional file 6: Figure S4. XTT assay revealed lack of significant proliferation effect on day 5 in ES-2 and OVCA420 cells stably transfected with ERβ5 as compared to control cells, whereas FAK inh 14 could inhibit OVCA420 basal cell proliferation on day 5. ns, not significant; **, *P* < 0.005. (PDF 81 kb)

## Abbreviations

ERs: Estrogen receptors
FAK: Focal adhesion kinase
SERMs: Selective estrogen receptor modulators
