# Supplementary material for: Differential expression of estrogen receptor subtypes and variants in ovarian cancer: effects on cell invasion, proliferation and prognosis
Source: BMC Cancer. 2017 Aug 31;17:606. doi: 10.1186/s12885-017-3601-1 (PMC5579953; doi:10.1186/s12885-017-3601-1)
Supplement: Supplementary file 1 — Primary antibodies used for immunohistochemistry and immunoblotting. (DOC 43 kb) [file 12885_2017_3601_MOESM1_ESM.doc]

**Table S1. Primary antibodies used for immunohistochemistry and immunoblotting.**

| **Target Protein** | **Animal source *** | **Catalog #** | **Applications ^**^** | **Vendor** |
| --- | --- | --- | --- | --- |
| ERα | Rabbit | AF1997 | IHC (1:50), IB (1:500) | Biocare (Concord, CA) |
| ERβ1 | Mouse | MCA1974 | IHC (1:20), IB (1:200) | Serotec (Raleigh, NC) |
| ERβ2 | Mouse | MCA2279 | IHC (1:100), IB (1:2000) | Serotec |
| ERβ5 | Mouse | MCA4676 | IHC (1:200), IB (1:2000) | Serotec |
| 6x-His Epitope Tag | Mouse | R930-25 | IB (1:1000) | Life technologies (Waltham, MA) |
| p-FAK | Rabbit | 8556 | IB (1:1000) | Cell Signaling (Beverly, MA) |
| FAK | Rabbit | sc-558 | IB (1:200) | Santa Cruz biotechnology, Inc (Santa Cruz, CA) |
| p-c-Src | Rabbit | 6943 | IB (1:1000) | Cell Signaling |
| c-Src | Rabbit | ab47405 | IB (1:1000) | Abcam (Cambridge, MA) |
| Actin | Rabbit | A5060 | IB (1:1000) | Sigma (St. Louis, MO) |
| α tubulin | Mouse | sc-8035 | IB (1:200) | Santa Cruz biotechnology, Inc |
| c-Jun | Mouse | 9165 | IB (1:1000) | Cell Signaling |

* Antibodies prepared in corresponding host animals.

**IHC: immunohistochemistry; IB: immunoblotting.
