# Supplementary material for: Differential expression of estrogen receptor subtypes and variants in ovarian cancer: effects on cell invasion, proliferation and prognosis
Source: BMC Cancer. 2017 Aug 31;17:606. doi: 10.1186/s12885-017-3601-1 (PMC5579953; doi:10.1186/s12885-017-3601-1)

Figure S1. Box plot showing comparison of the median nERβ1 immunoreactivity score in primary carcinomas versus matched metastatic foci.

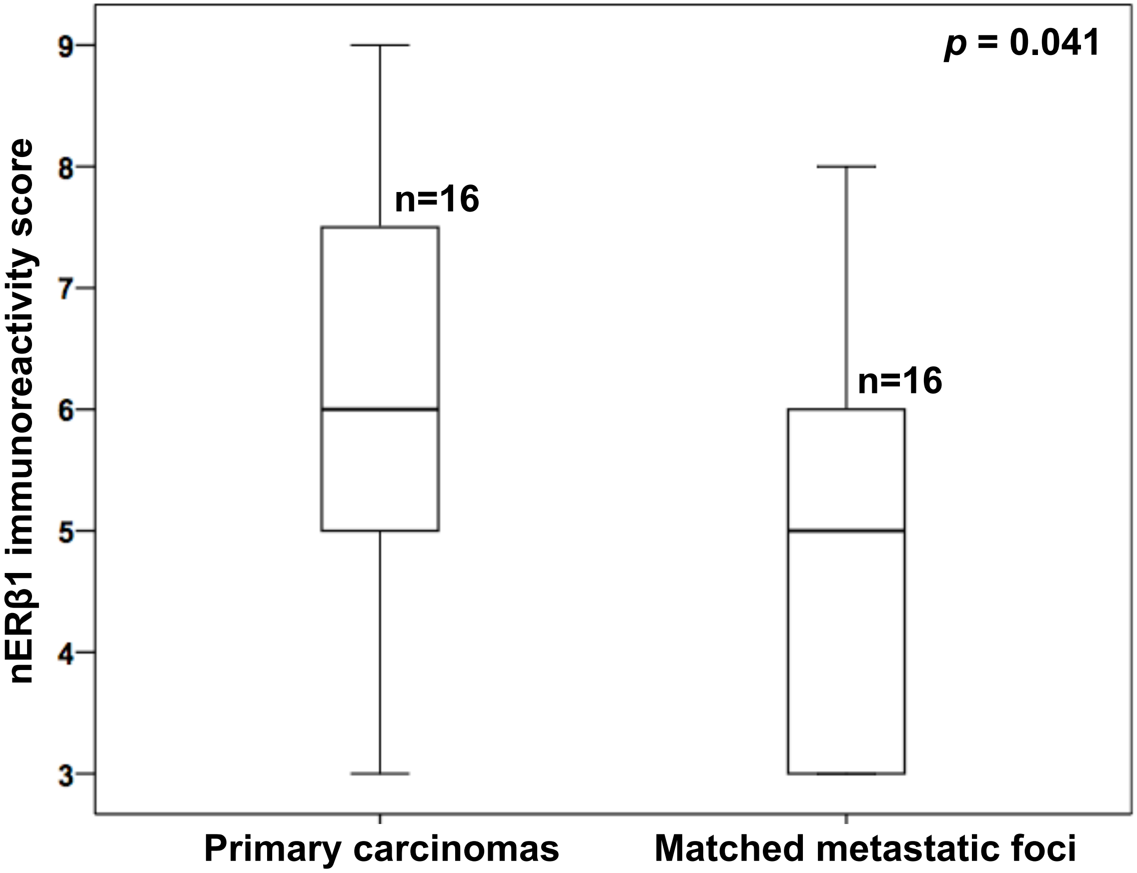

Supplement: Supplementary file 2 — Box plot showing comparison of the median nERβ1 immunoreactivity score in primary carcinomas versus matched metastatic foci. (PDF 69 kb) [file 12885_2017_3601_MOESM2_ESM.pdf]
