# Supplementary material for: Differential expression of estrogen receptor subtypes and variants in ovarian cancer: effects on cell invasion, proliferation and prognosis
Source: BMC Cancer. 2017 Aug 31;17:606. doi: 10.1186/s12885-017-3601-1 (PMC5579953; doi:10.1186/s12885-017-3601-1)
Supplement: Supplementary file 3 — Correlation coefficients between ERs expression in ovarian cancer. (DOC 55 kb) [file 12885_2017_3601_MOESM3_ESM.doc]

Table S2. Correlation coefficients between ERs expression in ovarian cancer.

|  | **Variables (n-90)** | **nERα** | **cERα** | **nERβ1** | **cERβ1** | **nERβ2** | **cERβ2** | **nERβ5** | **cERβ5** |
| --- | --- | --- | --- | --- | --- | --- | --- | --- | --- |
| **nERα** | Correlation Coefficient | - | 0.060 | -.150 | -0.026 | 0.068 | 0.147 | -0.014 | 0.085 |
|  | P-value |  | 0.574 | .158 | 0.809 | 0.524 | 0.167 | 0.899 | 0.424 |
| **cERα** | Correlation Coefficient | 0.060 | - | 0.100 | 0.111 | 0.017 | 0.041 | -0.041 | 0.123 |
|  | P-value | 0.574 |  | 0.922 | 0.300 | 0.876 | 0.701 | 0.705 | 0.249 |
| **nERβ1** | Correlation Coefficient | -0.150 | 0.100 | - | **0.305^**^** | 0.016 | -0.147 | **0.219^*^** | -0.154 |
|  | P-value | 0.158 | 0.922 |  | **0.003** | 0.884 | 0.166 | **0.038** | 0.147 |
| **cERβ1** | Correlation Coefficient | -0.026 | 0.111 | **0.305^**^** | - | 0.131 | **0.279^**^** | 0.168 | **0.357^**^** |
|  | P-value | 0.809 | 0.300 | **0.003** |  | 0.217 | **0.008** | 0.113 | **0.001** |
| **nERβ2** | Correlation Coefficient | 0.068 | 0.017 | 0.016 | 0.131 | - | 0.207 | 0.157 | -0.025 |
|  | P-value | 0.524 | 0.876 | 0.884 | 0.217 |  | 0.050 | 0.139 | 0.815 |
| **cERβ2** | Correlation Coefficient | 0.147 | 0.041 | -0.147 | **0.279^**^** | 0.207 | - | 0.048 | 0.172 |
|  | P-value | 0.167 | 0.701 | 0.166 | **0.008** | 0.050 |  | 0.653 | 0.105 |
| **nERβ5** | Correlation Coefficient | -0.014 | -0.041 | **0.219^*^** | 0.168 | 0.157 | 0.048 | - | 0.170 |
|  | P-value | 0.899 | 0.705 | **0.038** | 0.113 | 0.139 | 0.653 |  | 0.108 |
| **cERβ5** | Correlation Coefficient | 0.085 | 0.123 | -0.154 | **0.357^**^** | -0.025 | 0.172 | 0.170 | **0.304^**^** |
|  | P-value | 0.424 | 0.249 | 0.147 | **0.001** | 0.815 | 0.105 | 0.108 | **0.004** |

*, *P*<0.05; **, *P*<0.01. Those with significant P-values are shown in bold.
