# Supplementary material for: Differential expression of estrogen receptor subtypes and variants in ovarian cancer: effects on cell invasion, proliferation and prognosis
Source: BMC Cancer. 2017 Aug 31;17:606. doi: 10.1186/s12885-017-3601-1 (PMC5579953; doi:10.1186/s12885-017-3601-1)

Figure S2. Immunoblot analyses of ERβ2 and ERβ5 in OVCA420 cells stably transfected with His-tagged ERβ2, ERβ5 or control vector.

.

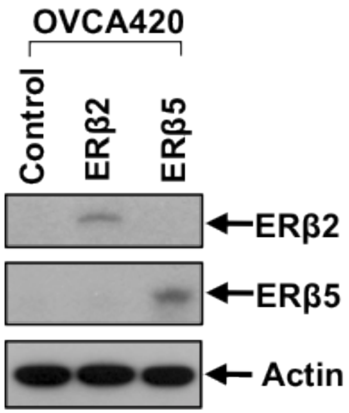

Supplement: Supplementary file 4 — Immunoblot analyses of ERβ2 and ERβ5 in OVCA420 cells stably transfected with His-tagged ERβ2, ERβ5 or control vector. (PDF 57 kb) [file 12885_2017_3601_MOESM4_ESM.pdf]
