# Supplementary material for: Differential expression of estrogen receptor subtypes and variants in ovarian cancer: effects on cell invasion, proliferation and prognosis
Source: BMC Cancer. 2017 Aug 31;17:606. doi: 10.1186/s12885-017-3601-1 (PMC5579953; doi:10.1186/s12885-017-3601-1)

Figure S3. Overexpression of ERβ2 increased ovarian cancer cell migration and invasion. (a) Immunoblot analyses of exogenous His-tagged ERβ2 in TOV-21G cells stably transfected with His-tagged ERβ2 or control vector. (b) *in vitro* migration and invasion assays in TOV-21G cells overexpressing ERβ2. Upper panel: representative images of migrating or invading cells. Lower panel: Cell migration or invasion presented as percentage of control; n=3; *, $p<0.05$.

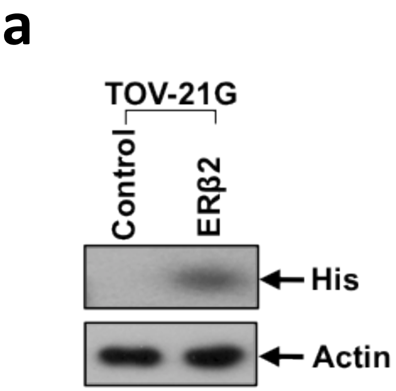

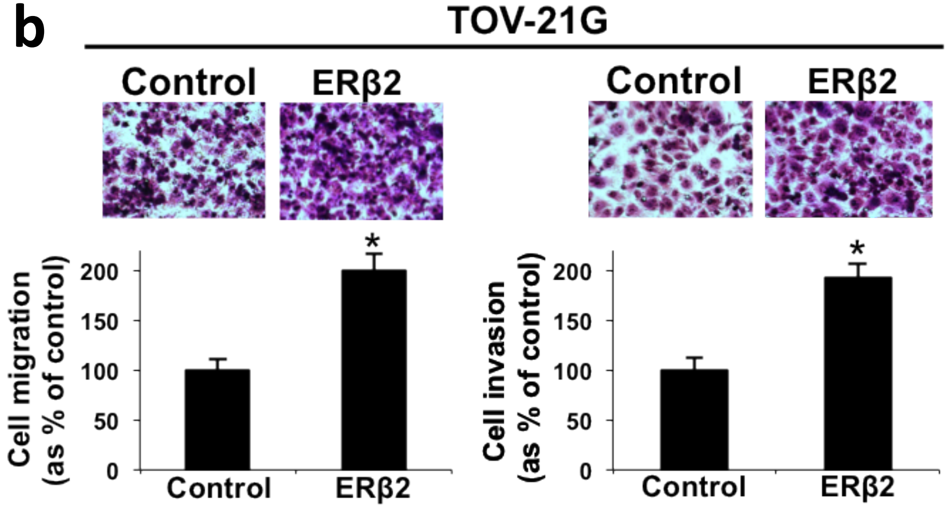

Supplement: Supplementary file 5 — Overexpression of ERβ2 increased ovarian cancer cell migration and invasion. (a) Immunoblot analyses of exogenous His-tagged ERβ2 in TOV-21G cells stably transfected with His-tagged ERβ2 or control vector. (b) In vitro migration and invasion assays in TOV-21G cells overexpressing ERβ2. Upper panel: representative images of migrating or invading cells. Lower panel: Cell migration or invasion presented as percentage of control; n = 3; *, p < 0.05. (PDF 223 kb) [file 12885_2017_3601_MOESM5_ESM.pdf]
