# Supplementary material for: Differential expression of estrogen receptor subtypes and variants in ovarian cancer: effects on cell invasion, proliferation and prognosis
Source: BMC Cancer. 2017 Aug 31;17:606. doi: 10.1186/s12885-017-3601-1 (PMC5579953; doi:10.1186/s12885-017-3601-1)

Figure S4. XTT assay revealed lack of significant proliferation effect on day 5 in ES-2 and OVCA420 cells stably transfected with ERβ5 as compared to control cells, whereas FAK inh 14 could inhibit OVCA420 basal cell proliferation on day 5. ns, not significant; **, *P*<0.005.

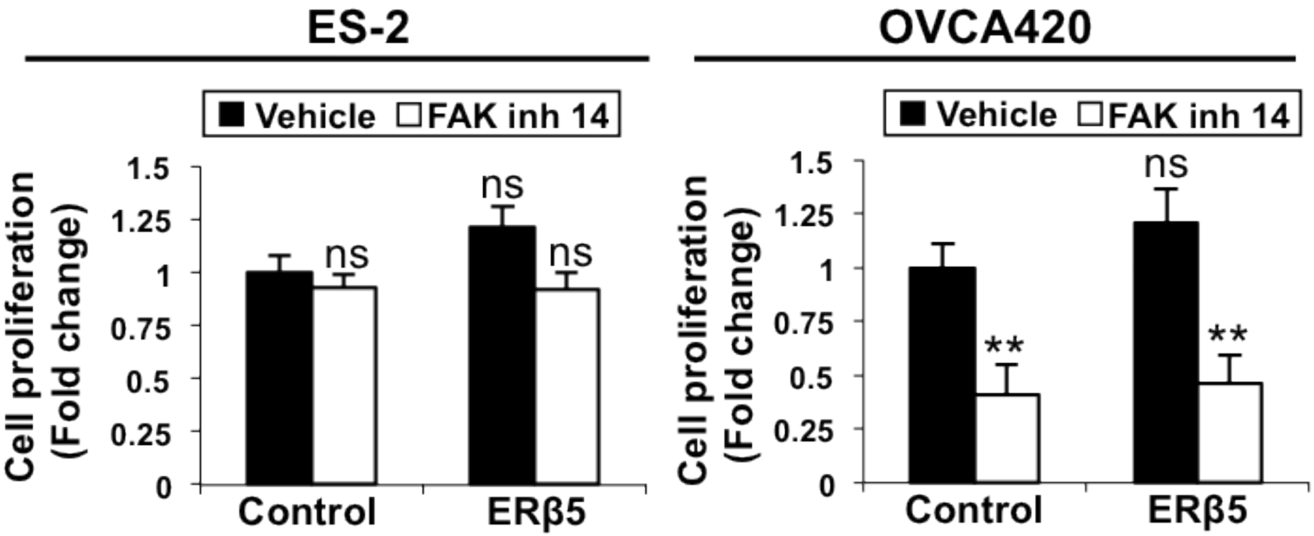

Supplement: Supplementary file 6 — XTT assay revealed lack of significant proliferation effect on day 5 in ES-2 and OVCA420 cells stably transfected with ERβ5 as compared to control cells, whereas FAK inh 14 could inhibit OVCA420 basal cell proliferation on day 5. ns, not significant; **, P < 0.005. (PDF 81 kb) [file 12885_2017_3601_MOESM6_ESM.pdf]
